# Persistent foot-and-mouth disease virus infection of the bovine nasopharynx is associated with suppression of innate and cellular immunity

**DOI:** 10.1371/journal.pone.0340425

**Published:** 2026-01-23

**Authors:** Benedikt Litz, Florian Pfaff, Leonie F. Forth, Sara Hägglund, Jean-François Valarcher, Martin Beer, Michael Eschbaumer

**Affiliations:** 1 Institute of Diagnostic Virology, Friedrich-Loeffler-Institut, Greifswald-Insel Riems, Germany; 2 Host Pathogen Interaction Group, Unit of Ruminant Medicine, Department of Clinical Sciences, Swedish University of Agricultural Sciences (SLU), Uppsala, Sweden; Central Laboratory for Evaluation of Veterinary Biologics, Agricultural Research Center, EGYPT

## Abstract

Foot-and-mouth disease is a devastating disease of cattle that is caused by foot-and-mouth disease virus (FMDV). After acute infection, FMDV persists in the upper respiratory tract of about 50% of infected cattle. The persistent infection is characterized by very localized viral replication in the absence of clinical signs, but the underlying mechanisms are still not clear. In our study, we investigated tissue samples collected from 20 cattle which had been experimentally infected with FMDV O/FRA/1/2001. In 17 animals, the infection persisted for longer than 28 days. Epithelial tissue from the dorsal nasopharynx and the dorsal soft palate (DSP), the two main locations for persistent infection, was collected at necropsy. Five biological replicates from each animal and location were screened by FMDV specific RT-qPCR, and subsets of the samples were selected for transcriptome sequencing (n = 52) and protein mass spectrometry (n = 18). There was a good correlation between the expression patterns identified by the transcriptomic and proteomic analysis. Higher loads of viral genome were detected in DSP samples. Overexpression of cellular markers for follicle-associated epithelium (FAE) and downregulated genes of epithelial integrity and keratinization correlated with viral genome loads, confirming the microanatomic localization of persistent FMDV infection in follicle-associated epithelium (FAE) in lymphoid tissue of the nasopharynx. An upregulation of genes which negatively influence T-cell responses indicates a T-cell exhaustion, most likely caused by prolonged immune stimulation. Moreover, decreased expression levels of *RIG-I* and *TRAF6* probably resulted in inhibited detection of viral RNA by the innate immune system and ultimately an impeded type I interferon response. These observations are in line with the hypothesis that FMDV actively suppresses the local immunity in FAE to maintain a persistent infection in the bovine nasopharynx.

## Introduction

Foot-and-mouth disease virus (FMDV; species *Aphthovirus vesiculae*, genus *Aphthovirus*, family *Picornaviridae*) infects mammals of the order Artiodactyla and causes severe outbreaks of vesicular disease in agriculturally important species such as cattle and pigs. Persistent FMDV infection follows the acute infection in roughly 50% of infected cattle. In contrast to fulminant foot-and-mouth disease (FMD) in the acute phase, no clinical signs are observed during persistent infection and onward transmission from persistently infected cattle is unlikely [1]. On the other hand, in persistently infected African buffalo (*Syncerus caffer*), which are the natural host of FMDV, transmission is common and probably contributes to maintain endemicity in buffalo populations in the wild [2]. To detect persistently infected animals, so-called “carriers”, oropharyngeal fluid (OPF) is collected using a probang cup. Probang sampling collects epithelial scrapings from the caudal nasopharynx, since persistent FMDV infection is restricted to epithelial surfaces in this otherwise inaccessible location [3].

The epithelial tissues of the dorsal nasopharynx (DNP) and the dorsal soft palate (DSP) have been identified as the main sites of persistent infection in cattle [4]. By using immunofluorescence and laser-capture microdissection (LCM), its microanatomical location was determined to be the follicle-associated epithelium (FAE) in this region [5]. The FAE is a specialized epithelial structure overlying the mucosa-associated lymphatic tissue (MALT), which is important for the induction of mucosal immunity. Sampling of antigens from the lumen and their transport to the subepithelial MALT is carried out by microfold cells (M-cells) [6]. These cells are found in similar epithelia overlying lymphoid follicles throughout the body, such as in the Peyer’s patches or the bronchial associated lymphoid tissue (BALT) [7]. Histologically, the FAE of the MALT in the nasopharynx of cattle differs from the surrounding epithelium by an incomplete base membrane and disorganized cuboidal epithelial cells interspersed with mixed mononuclear cells. Overall, FAE appears rarefied and disorganized [8].

The FAE has been the subject of a previous study to analyze the gene expression in specific epithelia of the nasopharynx using LCM, which found an inhibition of T-cell mediated immunity [5]. In other studies, a bovine whole-transcriptome microarray was used for the analysis of gene expression using tissue samples collected from persistently infected animals [9–11]. RNA sequencing (RNA-seq) as a method for comprehensive unbiased transcriptomic analysis has been applied to persistently infected cell lines from non-bovine species [12], to primary cultures of bovine tissue other than pharyngeal epithelium [12,13] and in an air–liquid interphase cell culture model prepared from bovine epithelial tissue from the DSP [14]. In this study, we used RNA-seq and mass spectrometry for the first time to investigate tissue samples collected from the DNP and the DSP of 17 persistently FMDV-infected cattle. The transcriptomic and proteomic analysis from the same samples showed high congruency between gene expression and protein abundance, mutually corroborating the results of each method.

The findings improve our understanding of the virus-host dynamics occurring during persistent infection and how the virus may be able to suppress innate immunity to maintain active replication in the epithelia of the nasopharynx despite a strong systemic immune response.

## Materials and methods

### Animal trial

A vaccination trial was carried out under BSL4vet conditions at the Friedrich-Loeffler-Institut on the Isle of Riems. Twenty Holstein-Friesian heifers (*Bos taurus*; 6–12 months) obtained from the same commercial herd were split into four groups: six animals were vaccinated twice intramuscularly (i.m.) at an interval of three weeks with a commercially available FMDV O_1_ Manisa vaccine, six animals were vaccinated three times intranasally with an experimental vector vaccine 21 and 52 days after the first vaccination, four animals were vaccinated three times i.m. with the same vector vaccine and one control group of four animals was injected i.m. with sterile PBS. The animals were challenged by intranasopharyngeal instillation [15] two weeks after the third vaccination (seven weeks after the second) with a twice plaque-purified isolate of FMDV O/FRA/1/2001 which had been passaged once in cattle after intraepidermolingual inoculation. The full genome sequence of the challenge virus is available in GenBank (accession no. OV121130.1). Clinical examination under xylazine sedation (0.3 mg/kg estimated body weight i.m.; reversed with 0.05 mg/kg atipamezole) was performed every day from 2 to 8 days post-challenge (dpc). During the acute phase analgesic treatment was performed in animals which developed lesions using meloxicam (0.5 mg/kg). The animals were euthanized in groups of four per day beginning on 35 dpc.

The cattle were brought into the BSL4vet facility 7 day before the beginning of the trial to accommodate to the new environment and stayed in the trial for 115 days. All staff working with the animals were educated to handle the animals carefully and reduce stress to a minimum. The temperature of the animals was measured daily and clinical appearance was scored using a clinical score sheet. The predefined humane endpoints for this trial were recumbency, loss of the claw, a clinical score over 10 or other appearances that suggest unacceptable suffering. No animals reached humane endpoints or died before the end of the trial. The protocol of the animal trial was approved by the State Office for Agriculture, Food and Fisheries of Mecklenburg-Vorpommern (LALLF M-V) under the file no. 7221.3-1-019/18.

### Sample collection

Serum and nasal fluid were collected every day for 8 days post challenge (dpc) and on 10, 14, 17, 21, 24, 28, 31, and 35 dpc. Probang samples were collected on 0, 14, 17, 21, 24, 28, and 35 dpc. Tissue samples from the nasopharyngeal region were collected at necropsy. To reflect the focal distribution of persistent FMDV infection in this region, five biological replicates were collected from each DNP and DSP epithelium. To avoid any degeneration of the mRNA, these samples were immediately frozen in liquid nitrogen and stored at −80°C.

### Virus isolation from probang samples

Sampling of OPF using a probang cup was performed to determine if animals were persistently infected with FMDV. The collected OPF was mixed with 4 ml of cell culture medium and homogenized by repeated aspiration using a 1.6 mm blunt cannula. Then, half of the sample was mixed with an equal amount of 1,1,2-trichloro-1,2,2-trifluoroethane (TTE) [3] to dissociate any bound antibody. Following vigorous shaking for 5 minutes and subsequent centrifugation at 1000 × g for 10 minutes at 4°C, the supernatant was removed and aliquoted. A 25 cm^2^ culture flask of 90% confluent LFBK-αVβ6 cells (porcine kidney cells expressing bovine αVβ6 integrin monolayer, CCLV-RIE 1419) [16] was then inoculated with 250 µl of the TTE-treated OPF solution. The remainder was stored at −80°C.

### RNA extraction

Disintegration of the tissue samples was performed with a CP02 cryoPREP (Covaris). The pulverized tissue was mixed with 250 µl AL buffer (Qiagen) and 750 µl TRIzol LS (Invitrogen). After removal from biocontainment, the samples were mixed with 200 µl trichloromethane and separated by centrifugation. Approximately 400 µl of the upper aqueous phase containing the RNA were removed and total RNA was extracted using the Agencourt RNAdvance Tissue Kit (Beckman Coulter) with a KingFisher Flex magnetic particle processor (Thermo Fisher Scientific). The quantity and quality of extracted total RNA were measured using a NanoDrop 1000 spectrophotometer (Thermo Fisher Scientific).

### FMDV genome detection

FMDV RNA was detected with an RT-qPCR using AgPath‑ID One‑Step RT‑PCR Reagents (Thermo Fisher Scientific) and a primer/probe set targeting the highly conserved 3D coding region [16].

### Library preparation and sequencing

From the 200 tissue samples, 52 were selected for transcriptomic analysis based on the results of the FMDV 3D RT-qPCR. These included 27 DNP and 25 DSP samples from 13 of the persistently infected animals and from the three animals which had cleared the infection before 28 dpc (S1 Table). From the extracted total RNA, mRNA was isolated using the Dynabeads mRNA DIRECT Micro Purification Kit (Invitrogen) and the Colibri Stranded RNA Library Prep Kit for Illumina (Invitrogen) was used for library preparation. ERCC internal control (Invitrogen) was used before mRNA extraction as recommended by the manufacturer. In detail, isolated mRNA was fragmented by RNase III to an approximate length of 150 nucleotides. Adapters were hybridized and then ligated to the fragmented RNA. Adapter-ligated RNA was transcribed into cDNA using 10 × SuperScript IV Enzyme Mix and purified. Finally, cDNA was amplified in 12 or 13 cycles (depending on the input) using appropriate index primers for the generation of barcoded Illumina libraries. Length and quality of the libraries was assessed on an Agilent 4150 TapeStation (Agilent Technologies). The libraries were quantified using a Qubit 2.0 (Invitrogen) and the Qubit dsDNA HS Assay Kit (Invitrogen) and pooled at an equimolar ratio. For sequencing, a NovaSeq machine (Illumina) running in 100 bp single-end mode was used.

### Mass spectrometry

For proteomics analysis, 18 tissue samples, which had also been used for RNAseq, were sent to Proteome Sciences (Frankfurt, Germany). Protein was extracted from the organic TRIzol phase, tryptic peptides were produced, labelled with 18 isobaric TMTpro reagents (Thermo Fisher Scientific) and combined to generate one TMTpro 18-plex sample. The 18-plex was fractionated into 24 fractions using basic reversed-phase chromatography. All fractions were analyzed by tandem mass spectrometry using a data-dependent acquisition method combined with an inclusion list for peptides expected to derive from FMDV. Peptide quantification was based on TMTpro reporter ion intensities, and a proprietary statistical pipeline was used to determine regulated peptides and proteins.

Statistical analysis was conducted using internally developed scripts written in the R statistical programming language [17]. All tools were developed to work with TMT-labelled MS data, and include functionality for dealing with isolation interference, isotopic crosstalk, PSM normalisation and summarisation into peptides and proteins. The pre-processing and statistical testing tool includes dedicated filtering, data normalisation and peptide summarisation functionality [18,19]. The thresholds were set at a *p-value* of 0.0001 and a log2FC of ±0.58. All tools were internally verified, provide configuration and log files, and their runs can be reproduced at any other time.

### Statistical analysis of gene expression data

Raw reads were initially trimmed by removing low quality regions and adapter contamination using TrimGalore (v0.6.6) together with cutadapt (v1.18) running in automated adapter detection mode. A genome and transcriptome reference for cattle was received from NCBI (ARS-UCD1.2; GCA_002263795.2) and combined with reference sequences for ERCC internal controls and the FMDV O/FRA/1/2001 inoculum sequence. The RNA transcriptome and DNA genome references were concatenated into a single file and a decoy-aware index was created using the “index” function of Salmon (v1.9.0). The index was then used to quantify the transcript abundancies within each sample using the “quant” function of Salmon. Corrections for sequence-specific bias, fragment-level GC bias and position-specific fragment bias were activated and 10 bootstraps were applied in order to compute abundance estimates. The resulting transcript abundancies were further analyzed using R (v4.3.1) [17] and imported into a DESeq2 (v1.40.2) compatible format using the package tximport (v1.28.0). The transcripts in the dataset were filtered based on their abundancy, so that only transcripts that scored a relative abundancy of at least 10 transcripts per million (TPM) observed within at least 2 independent samples were further analyzed. Before principal component anaylsis (PCA), the filtered abundancy data was transformed using the “rlog” function from the “DESeq2” package that applies a regularized log transformation. PCA was done using the “prcomp” function from the stats package based on the transformed abundancies of 1000 transcripts that showed the highest variance within the dataset.

In order to find differentially expressed genes (DEG), we applied the main “DESeq” function from DESeq2 package using different designs in order to reflect the underlying contrast. The resulting log2 fold changes (log2FC) were further adjusted using the function “lfcShrink” function from the DESeq2 package. The final dataset was filtered using a cutoff *p-value* of <0.05 and a cutoff log2FC of>|1|.

### Pathway analysis

DE gene sets were analyzed for their biological function using the “enrichPathway” function in the “reactomePA” package (version 1.46.0) [20]. An overview of enriched pathways was conducted using the g:Profiler webservice (https://biit.cs.ut.ee/gprofiler/gost) [21]. Predicted networks and Canonical Pathways were generated using Qiagen Ingenuity Pathway Analysis (IPA) software (version 90348151) [22].

### Statistics

All statistical analyses were performed using R (version 4.3.1) [17]. The binomial proportion confidence interval for the incidence of persistent infection was calculated by the Wilson method. Group-wise differences (between the vaccination groups) in the amount of detectable FMDV RNA in the tissue samples (represented by the Cq value in the 3D RT-qPCR) were examined with a one-way analysis of variance. Group-wise differences in the amount of detectable FMDV RNA in the tissue samples (represented by the Cq values obtained by 3D RT-qPCR) were assessed using a Wilcoxon rank-sum test (“wilcox.test” function from the “stats” package), as the data did not meet the assumptions of normality required for parametric testing. Association between expression of specific FAE-associated genes and FMDV presence was analysed using Spearman correlation. In detail, regularized log transformed gene counts and Cq values from the FMDV-specific 3D RT-qPCR were correlated using the functions “cor” and “cor.test” from the “stats” package.

## Results and discussion

### Clinical protection and incidence of persistent infection

Animals vaccinated with the experimental vaccine were not protected from challenge and developed clinical FMD with vesicular lesions similar to those observed in non-vaccinated animals, while commercially vaccinated cattle did not show any clinical signs. However, the efficacy of the vaccines and the outcome of the challenge infection is outside of the scope of the study reported here.

Virus isolation from OPF samples was performed to determine the incidence of persistent infection. Carriers are defined as cattle from which virus can be recovered later than the 28^th^ day of infection [23]. By this definition, there were 17 persistently infected carrier animals and the incidence of persistent FMDV infection was 85% (confidence interval: 64–95%). OPF from two animals (426 and 662) remained negative throughout the entire trial. Animal 426 had been vaccinated with the commercial vaccine and did not show any clinical signs after challenge. Animal 662, on the other hand, had been vaccinated intramuscularly with the experimental vaccine and did develop clinical FMD. Probang samples from one animal (940) were positive in the virus isolation on 21 and 24 dpc, but not thereafter; hence, it was not considered a carrier. OPF from two animals (ear tags 506 and 508) was positive in the virus isolation on day 28 and before, but not on day 35.

Despite the complete clinical protection of the commercial vaccine, it did not prevent persistent FMDV infection in 5 of the 6 vaccinated animals (except 426, as mentioned above). The high observed carrier incidence lies above the commonly assumed value of roughly 50% [1], but this may be serotype- or even strain-dependent as experiments with SAT1, 2 and 3 in African buffalo have indicated [2].

### FMDV tissue distribution and overall gene expression

**Fig 1:**
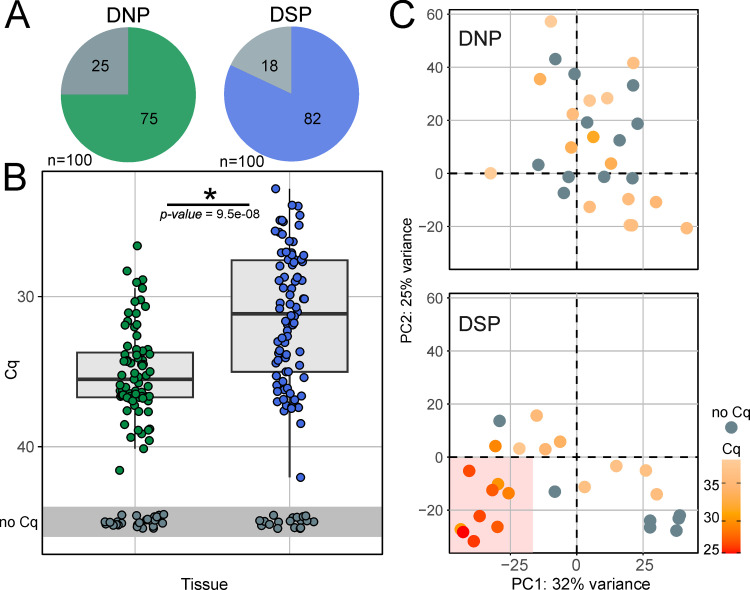
Tissue sample characteristics. **(A)** Ratio of FMDV RT-qPCR positive samples for DNP (green) and DSP (blue) samples. **(B)** Boxplot showing FMDV-specific RT-qPCR Cq values as correlate for viral load in DNP and DSP samples. Cq values for both tissue types were compared using the Wilcoxon rank-sum test. **(C)** Principal component analysis (PCA) of gene expression in selected DNP and DSP samples (n = 52) based on the normalized transcript abundancies of the 1000 transcripts with the highest variance within the dataset. The PCA illustrates the overall variance structure of the dataset, with PC1 and PC2 capturing the major axes of separation among samples. FMDV-specific RT-qPCR Cq values are indicated by color. A distinct cluster of highly FMDV-positive DSP samples is highlighted by a red area.

The entire sample set consisted of 200 tissue samples collected from 20 animals, of which 17 were persistently infected. In this sample set, 100 samples were from the DSP and 100 from the DNP. Among DSP samples, a higher proportion was positive in the RT-qPCR for FMDV compared to DNP samples (Fig 1A). DSP samples also contained significantly higher loads of FMDV genome than DNP (Fig 1B). The median quantification cycle (Cq) value was 31.1 for positive DSP samples and 35.5 for positive DNP samples. The vaccination status of the animals had no influence on the quantity of detectable FMDV RNA in the tissues collected at 35 dpc (see S1 Table). Additionally, in a principal component analysis (PCA) based on normalized gene expression data from the 27 DNP and 25 DSP tissue samples that had been selected for the transcriptomic analysis, a cluster of highly positive DSP samples (Cq ≤ 30) was visible (Fig 1C). In the DNP, on the other hand, no clustering by Cq value was observed, as these samples contained overall lower viral genome loads than DSP samples (Fig 1B, C). This implies a tissue preference of persistent infection and the clustering of highly positive DSP samples in the PCA suggests that samples with a high viral genome load have similarly modulated host gene expression. In contrast to the FMDV RNA content of the tissue samples, the vaccination status was not reflected in the clustering of samples in the PCA.

Presence of viral RNA in the epithelia of the bovine nasopharynx of persistently infected animals has been previously shown by in-situ hybridization by the authors [24] and others [25,26], and viral antigen was observed by fluorescence microscopy [5]. Virus isolation from tissues from these locations was performed in some of these studies as well [5,24]. In the present study, the homogenization of the tissue samples in lysis buffer did not allow microscopy or virus isolation.

### Overall differential gene expression

As there were only two animals in the trial that were unequivocally not persistently infected (426 and 662), we were not able to base our comparison on the carrier state of the animals themselves and had to classify the individual tissue samples instead. From the set of tissue samples, 27 DNP and 25 DSP samples were selected for transcriptomic analysis using RNA-seq. The selection was based on the Cq value in the FMDV RT-qPCR (S1 Table). The samples were assigned to one of three categories: high FMDV-positive (Cq ≤ 30), low FMDV-positive (Cq > 30) and FMDV-negative (no Cq). We tried to include both positive and negative samples from the same animals to reduce the impact of between-animal differences on the transcriptomic analysis.

Differential expression analysis across the defined sample contrasts revealed substantial variation in the number of differentially expressed genes (DEGs) depending on FMDV status and sampling context (**Table 1**). When all DSP samples were compared with all DNP samples, 294 genes were found to be differentially expressed. Restricting the comparison to FMDV-negative samples markedly reduced this number to 13, indicating that much of the difference in gene expression between sampling sites is correlated to the presence of viral RNA. Comparisons involving low-positive samples, whether from DSP or DNP, showed no differential expression relative to FMDV-negative samples, suggesting that low viral loads have a minimal impact on transcription. In contrast, the comparison focusing on highly FMDV-positive DSP samples yielded a robust response, with 413 DEGs identified (**Table 1**). As this contrast captured the clearest virus-associated gene expression signature, it was selected for subsequent analysis. For a summary of all detected DEGs see S2 Table.

**Table 1:** The number of differentially expressed genes (DEG) for different contrasts.

Contrast	Number of replicates	Number of total DEGs (up/down)
DSP ↔ DNP	25 ↔ 27	294 (168/126)
DSP+FMDV_neg_ ↔ DNP+FMDV_neg_	7 ↔ 11	13 (5/8)
DNP+FMDV_lowpos_ ↔ DNP+FMDV_neg_	16 ↔ 11	0
DSP+FMDV_lowpos_ ↔ DSP+FMDV_neg_	10 ↔ 7	0
DSP+FMDV_highpos_ ↔ DSP+FMDV_neg_	8 ↔ 7	413 (240/173)

“FMDV_highpos_” are samples with a Cq ≤ 30 (high positive), “FMDV_lowpos_” are samples with a Cq > 30 (low positive) and “FMDV_neg_” are FMDV-negative samples (no Cq) in the FMDV-specific RT-qPCR

A pathway analysis of these 413 DEGs showed that many of the upregulated genes are related to immune responses (for example *IL10*, *NLRC3*, *CCL20*, *TIMD4* and *CXCL8*), while many of the downregulated genes are associated with epithelial differentiation and keratinization (for example *KRT3*, *KPRP*, *CDSN*, *LCE3C*, LOC104971496 and *CWH43*). Similar to findings by Eschbaumer et al. and Zhu et al., several pathways related to the recruitment of leukocytes from the blood to peripheral tissues were upregulated. Both studies observed upregulated pathways of cellular metabolism and differentiation, but neither showed such strong downregulation of the pathway of keratinization [9,10]. A selection of the most significant differentially expressed canonical pathways is depicted in S1 Fig. The top up- and downregulated genes can be found in **Table 2**. In the following chapters, the DEGs from highly FMDV-positive DSP samples compared to FMDV-negative DSP samples were further analysed in detail. Therefore, we compared only DSP tissues grouped by their FMDV RNA content, but all samples derive from experimentally infected animals and the carrier status of the animals themselves was not taken into account.

**Table 2:** Top 10 up- and downregulated genes when comparing highly FMDV-positive (Cq ≤ 30) to negative DSP samples.

Gene	Name	Reactome pathways	*p-value*	log2FC
LOC112449072	multidrug resistance-associated protein 4-like		1.75E-02	10.39
*CD300LB*	CD300 molecule-like family member b	Immunoregulatory interactions between a lymphoid and a non-lymphoid cell	2.90E-02	6.86
*ELL3*	elongation factor for RNA polymerase II 3	RNA polymerase II transcribes snRNA genes	3.21E-04	6.04
*CXCL8*	C-X-C motif chemokine ligand 8	Signaling by interleukins	3.88E-02	6.02
LOC101905293	fibrous sheath-interacting protein 2-like		4.24E-05	5.23
*IL10*	interleukin 10	Signaling by interleukins	3.88E-02	5.12
*NLRC3*	NLR family CARD domain containing 3	IRF3-mediated induction of type I interferon	1.58E-03	5.11
*DSB*	MHC class II antigen DS beta		1.85E-02	5.07
*GPRIN3*	GPRIN family member 3	Assembly and cell surface presentation of NMDA receptors	7.83E-04	4.93
LOC112443438			1.94E-02	4.92
LOC282685	Bovine Rhesus-like protein		8.39E-03	−9.66
LOC100848804	small proline-rich protein 2E-like		2.47E-04	−8.65
*PNLIPRP3*	Pancreatic Lipase Related Protein 3	Digestion of dietary lipid	3.19E-02	−8.58
*KRT3*	keratin 3	Keratinization	2.04E-09	−8.36
LOC104971496	late cornified envelope protein 3B-like	Keratinization	3.25E-02	−8.34
*KPRP*	keratinocyte proline rich protein	Keratinization	4.71E-04	−7.97
*CDSN*	corneodesmosin	Keratinization	1.41E-03	−7.91
*UGT1A6*	UDP glucuronosyltransferase 1 family, polypeptide A6	Biological oxidations	1.36-03	−7.56
*LCE3C*	late cornified envelope 3C	Keratinization	5.73E-03	−7.38
*CALHM1*	Calcium homeostasis modulator protein 1	Sensory perception	1.75E-02	−7.22

The corresponding Reactome pathways are listed where possible. Positive log2 fold change (log2FC) indicates upregulation while negative log2FC indicates downregulation

### Consistent signatures across protein and transcript levels

**Fig 2:**
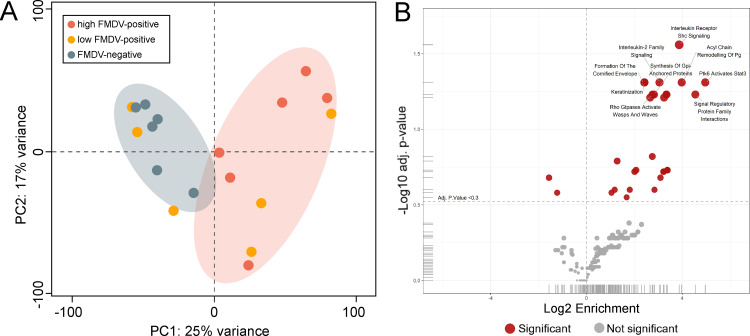
Proteomic analysis. **(A)** Principal component analysis (PCA) performed using normalized protein abundance values from the proteomic analysis of DSP samples. The PCA illustrates the overall variance structure of the dataset, with PC1 and PC2 capturing the major axes of separation among samples. Samples are grouped according to their FMDV Cq value: high FMDV-positive (Cq ≤ 30) in red, low FMDV-positive (Cq > 30) in orange and FMDV-negative (no Cq) in blue-gray. **(B)** Analysis of enriched Gene Ontology Biological Process (GOBP) terms derived from proteins differentially expressed between high FMDV-positive and FMDV-negative DSP samples. Each point represents a pathway term, plotted by log2 enrichment on the x-axis and –log10 adjusted p-value on the y-axis. Positive log2 enrichment indicates upregulation of pathways, negative log2 enrichment indicates downregulation. Red points denote pathways with statistically significant enrichment, whereas grey points represent nonsignificant terms.

The 18 samples selected for proteomic analysis corresponded to the DSP tissues used for RNA-seq. After the application of the TMTpro MS2 workflow, 79,888 peptides and 8,937 protein groups were quantified and assigned to the *Bos taurus* genome. Principal component analysis (PCA) of the 18 samples showed a clear separation of highly positive and negative samples along the 1^st^ dimension, accounting for 24.8% of the variance, while weakly positive samples were scattered between those two groups (Fig 2A). For samples 180 and 129, the rather low Cq values between 30 and 31 (barely above the cut-off for a classification as highly positive) may explain their clustering with the strongly positive samples.

Pronounced differences in protein abundance were observed between highly FMDV-positive (Cq ≤ 30) and FMDV-negative samples, as it has been also observed in the PCA of the transcriptomic data of the DSP samples. The following analysis will focus mainly on this contrast. In this group, 562 proteins were significantly regulated, of which 322 were downregulated and 240 upregulated. **Table 3** shows the 10 most upregulated and downregulated proteins. No viral proteins of FMDV were detected by mass spectrometry, presumably due to the lower sensitivity of this method compared to RNA detection by RT-qPCR.

**Table 3:** Top 10 list of significantly up- and downregulated characterized proteins when comparing highly FMDV-positive DSP samples to negative samples.

Protein	Name	*p-value* protein	log2FC protein	*p-value* mRNA	log2FC mRNA
LIMD2	LIM domain-containing protein 2	2.8E-04	2.16	6.6E-07	3.69
SCIMP	SLP adaptor and CSK interacting membrane protein	6.7E-04	2.11	2.3E-05	3.98
CXCL13	C-X-C motif chemokine	4.9E-06	2.01	3.8E-04	1.58
PLA2G2A	Phospholipase A2, membrane associated	2.8E-05	1.96	1.9E-02	2.23
IL4I1	Interleukin 4 induced 1	5.0E-04	1.56	5.7E-04	2.34
BOLA-DRB2	Ig-like domain-containing protein	2.2E-04	1.56	3.7E-03	1.88
MS4A1	Membrane spanning 4-domains A1	5.6E-04	1.50	8.7E-04	3.75
GIMAP7	GTPase, IMAP family member 7	3.6E-04	1.48	3.4E-04	1.99
FCRLA	Fc receptor like A	3.1E-04	1.47	1.7E-02	2.37
GRAP	GRB2-related adapter protein	4.4E-04	1.44	2.9E-06	3.16
LY6D	Lymphocyte antigen 6D	2.1E-05	−3.02	2.5E-03	−2.83
SBSN	Suprabasin	8.7E-06	−2.89	4.5E-08	−6.40
F1N6D1	WAP domain-containing protein	8.0E-05	−2.57	5.5E-05	−2.47
AKAP14	A-kinase anchoring protein 14	5.0E-05	−2.53	1.2E-06	−3.80
SPRR2F	Small proline-rich protein 2I-like	8.5E-07	−2.36	3.6E-07	−4.63
KLK13	Kallikrein related peptidase 13	2.8E-11	−2.35	1.3E-03	−2.01
KLK12	Kallikrein related peptidase 12	3.1E-08	−2.23	2.4E-05	−4.50
FAM25A	Protein FAM25A	6.6E-04	−2.15	n.s.	n.s.
SCEL	SCEL protein	1.4E-05	−2.13	n.s.	n.s.
A2ML1	Alpha-2-macroglobulin like 1	2.4E-04	−2.11	n.s.	n.s.

Selected and sorted by log2FC, filtered for peptide spectral matches (PSM) >1. A positive log2FC indicates upregulation, a negative log2FC downregulation. The results for the corresponding mRNAs are included for comparison. n.s.: not significant

A functional analysis indicated the enrichment of pathways associated with interleukin signaling, lipid metabolism, and keratinization, as well as Gene Ontology Biological Process (GOBP) terms associated with proteolysis, immune response and de-ubiquitylation (Fig 2B).

Overall, we detected 102 proteins to be significantly regulated in the proteomics analysis, which were already differentially expressed in the transcriptomics analysis. Among them, 101 showed concurring regulation, only one protein, PGLYRP2, was divergently regulated between the proteomic and transcriptomic analysis. This molecule is expressed in epithelial cells and has a positive effect on the recruitment of T regulatory (T_reg_) cells [27]. For 7 of the 20 highest up- or downregulated proteins identified in the proteomic analysis (SBSN, KLK12, FAM25A, A2ML1, LIMD2, SCIMP, GRAP), the corresponding gene was found to be regulated in the same way by RNA-seq.

### High viral load of persistent FMDV is associated to FAE markers

**Fig 3:**
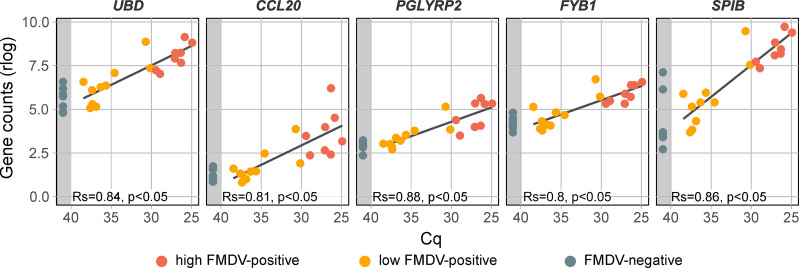
Gene expression of follicle-associated epithelium marker genes in DSP samples. Samples are grouped by their FMDV Cq value into highly positive (Cq ≤ 30, red), weakly positive (Cq > 30, orange) and negative (no Cq, grey area). Gene counts were normalized using a regularized log transformation (rlog) and the result of a linear regression is shown. Correlation between normalized gene counts and FMDV genome load was assed using Spearman’s rank correlation coefficient (Rs). Rs ranges from −1 to 1 and values closer to 1 or −1 indicate a strong positive or negative monotonic relationship, respectively. Rs = 0 indicates no monotonic association. A correlation was considered statistically significant if the *p-value* was less than 0.05.

The FAE has a different cellular phenotype in comparison to surrounding epithelia and can be observed microscopically. Gene expression of the specialized FAE tissue has been characterized well in Peyer’s patches in the intestinal tract [28,29]. Some of the previously reported FAE-specific marker genes (*UBD*, *CCL20*, *PGLYRP2*, *FYB1* and *SPIB)* were either significantly upregulated in the highly FMDV-positive DSP samples or their relative expression was significantly positively correlated to the RT-qPCR detected viral load (**Fig 3**) [28–30]. This could be interpreted as colocalization of FMDV genome and tissue with high FAE content. This is in accordance with previous immunofluorescence studies that localized the persistent FMDV infection in the FAE of the bovine nasopharynx, especially in the region of the caudal DSP [5].

Furthermore, the epithelium overlying the follicular dome has been described as “rarefied and disorganized” [8]. This could be reflected in our samples by strongly downregulated genes coding for structural constituents of the cornified epidermis (e.g., *LORICRIN*, *SERPINB12*, *KRT1*, *KRT10*, *KLK7*, *KLK6*) and components of the extracellular space (e.g., *ARG1*, *SERPINB12*, *AHSG*, *KRTDAP*, *KLK7*, *A2ML1*, LOC519132, *IL36A*, *IL36G*), some of which were among the 10 most strongly downregulated genes overall as shown in **Table 2**. This is reflected by the strong downregulation on the protein level of SBSN, which is involved in keratinization [31], and A2ML1, a component of the extracellular matrix. Fully differentiated keratinocytes usually express antimicrobial components such as the late cornified envelope (LCE) proteins [32]. Two of the genes coding for LCEs, *LCE3C* and LOC104971496 (bovine ortholog of *LCE3B*), were among the most downregulated genes (see **Table 2**). Since FAE is not a keratinized stratified squamous epithelium like most pharyngeal epithelia, its keratinocytes are not highly differentiated and the epithelial integrity is compromised to allow mononuclear cells to infiltrate into the epithelium to target invading pathogens. This results in a gene expression pattern like it was observed in this and previous studies [33].

In contrast to analyses with tissues prepared by LCM [5], using whole pieces of tissue provides a larger amount of higher-quality RNA, but does not allow any control over how much of the sample is FAE rather than normal nasopharyngeal epithelium. Using the level of detectable FMDV RNA to group these samples for further analysis will then lead to comparisons of non-infected normal nasopharyngeal epithelium with FMDV-infected FAE. The differential expression of genes coding for elements of keratinization or structural components of the extracellular matrix observed in this and previous studies [26] is likely due to this difference in sample composition.

### Sites of FMDV persistence show impaired desquamation

**Fig 4:**
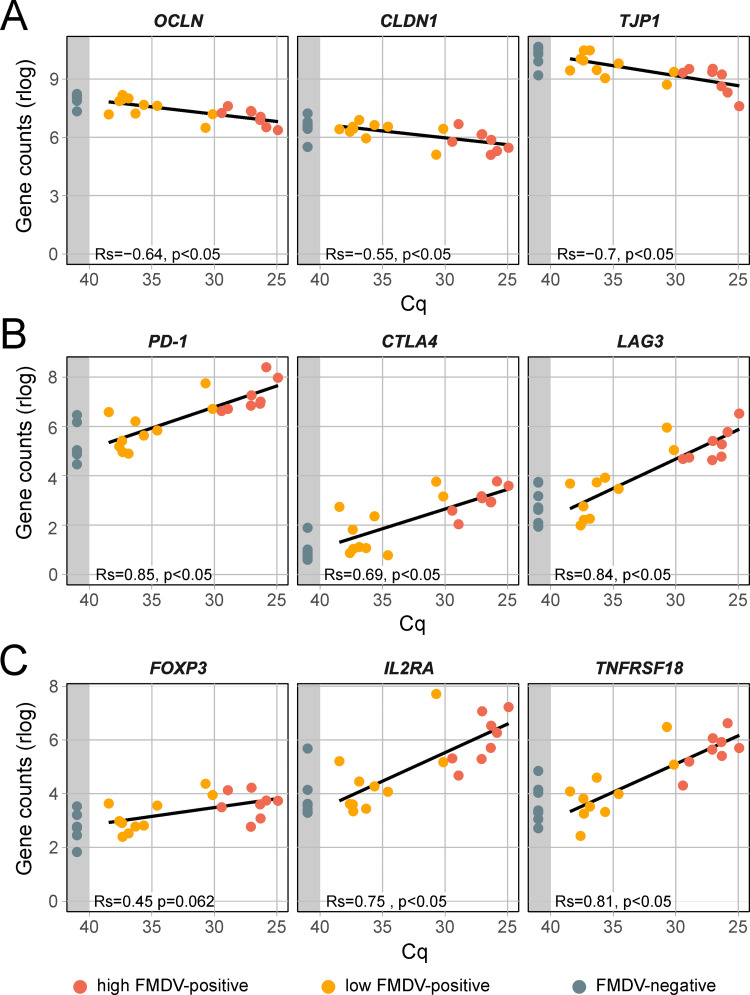
Gene expression of tight junction components, T-cell proliferation and markers for regulatory T cells in DSP samples. **(A)** Gene expression of the components of cellular tight junctions occludin (*OCLN*), claudin-1 (*CLDN1*) and the tight junction protein 1 (*TJP1*/*ZO-1*). **(B)** Gene expression of important immune checkpoints for regulating T-cell proliferation: *PD-1*, *CTLA4* and *LAG-3*. **(C)** Gene expression of marker genes for T_reg_ cells. Samples are grouped by their FMDV Cq value into highly positive (Cq ≤ 30, red), weakly positive (Cq > 30, orange) and negative (no Cq, grey area). Gene counts were normalized using a regularized log transformation (rlog) and the result of a linear regression is shown. Correlation between normalized gene counts and FMDV genome load was assed using Spearman’s rank correlation coefficient (Rs). Rs ranges from −1 to 1 and values closer to 1 or −1 indicate a strong positive or negative monotonic relationship, respectively. Rs = 0 indicates no monotonic association. A correlation was considered statistically significant if the *p-value* was less than 0.05.

Desquamation of superficial cells is typical of epithelia and has also been documented in lymphatic epithelia covering the tonsils [34]. To allow the shedding of cells, corneodesmosomes are cleaved by a set of different kallikreins [35]. In our FMDV-positive samples, genes coding for components of the corneodesmosome (e.g., *CDSN*, *DSG1*, *DSC2*, *DSC3*) but also several kallikreins (e.g., *KLK6*, −*7*, −*8*, −*12*, −*14*) were significantly downregulated. Especially the kallikreins 6, 7 and 12 are usually highly expressed in healthy lymphatic tissue of tonsils [36]. On the protein level, kallikrein-related peptidases (KLK7, −10, −11, −12, −13, −14) were among the proteins with the most pronounced change in abundance as well. As additional evidence of impaired desquamation, several inhibitors that usually control kallikreins, such as serpins (*SERPINB10*, *SERPINB12*) [37], α2 macroglobulins (*A2ML1*) [38], Kazal-type inhibitors (*SPINK5*, *SPINK7*) [39] and *AHSG* [40] were also downregulated in the FMDV-positive samples. The gene expression levels of the genes mentioned here are shown in S3 Table. The genes that code for components of tight junctions, such as occludin (*OCLN*), claudin-1 (*CLDN1*) and tight junction protein 1 (*TJP1*), were not significantly downregulated in highly FMDV-positive DSP samples. However, they were statistically significantly negatively correlated with FMDV genome load (Fig 4A). An absence of tight junctions can enhance paracellular viral spread in the epithelium and increase the receptor availability for FMDV [33].

### Apoptosis

A programmed cell death can be triggered in order to eliminate dysfunctional cells or limit viral replication [41]. In the pathway analysis, 28 genes related to apoptosis were upregulated in persistently FMDV-infected DSP, but careful consideration of their individual functions shows a more ambiguous picture. Some of these genes are associated with positive regulation of apoptosis such as the highly upregulated genes *ELL3* [42], *MMP9* [43] and *IKZF3* [44]. However, *ELL3*, which is the most strongly upregulated gene (see **Table 2**), has divergent functions. Its overexpression can promote apoptosis, but ELL3 has also been demonstrated to degrade the regulator of apoptosis p53 (TP53) and inhibit apoptosis in mouse embryonic stem cells [45]. In our study, p53 was not significantly upregulated, neither on the gene nor on the protein level. Another important inhibitor of p53 is BCL2 (*BCL2A1*) [46], which was significantly differentially expressed in FMDV-infected tissue. Viral orthologues of BCL2 were discovered in several DNA viruses such as African swine fever virus or lumpy skin disease virus, preventing premature cell death [47]. Together with the cellular inhibitor of apoptosis NAIP, BCL2A1 exerts a strong influence on the downstream apoptosis signaling [48], predicted to suppress several mechanisms leading ultimately to cell death (S2 Fig**).** In addition to this important regulator of programmed cell death, a few genes associated with anti-apoptotic features were upregulated in FMDV-infected tissues, including *IL10* [49] and *LRRK2* [50]. A tissue-specific aspect of apoptosis is the aforementioned insignificant upregulation of *SPIB*, which is necessary for the differentiation of M-cells and expressed in the FAE of MALT [29], but *SPIB* also inhibits detachment-induced apoptosis [51].

### Adaptive immunity

#### B-cell response.

The humoral response initiated by B-cells is an adaptive immune response in animals. In the blood, the primary response with an early onset of IgM followed by IgG is important for the clearance of viremia. In carrier animals, a prolonged IgA response in saliva can be observed [52]. In this study, the upregulation of *BCL7A*, which is restricted to B cells of tonsils and lymph nodes [53], suggests the presence of B cells and supports the findings from above, that the infected tissue is the FAE overlying the lymph follicles. Several genes of the B-cell activation pathway were significantly upregulated in FMDV-infected samples (*IL10*, *CTLA4*, *CD22*, *SH3KBP1*, *POU2F2*, *BANK1*). Of these, the *IL10* gene showed the highest differential expression. *CTLA4*, which is expressed by T_reg_ cells, inhibits B cell responses and decreases antibody levels [54], which seems contradictory to the higher antibody levels found in carriers. Furthermore, *CD22*, which was also upregulated, is mainly restricted to B cells and negatively regulates B-cell receptor signaling [55], but at the same time it is an adhesion molecule, which can regulate homing of antibody-producing B cells. The ligand for *CD22*, *St6Gal1*, is selectively expressed in the Peyer patches [56], which are a lymphatic epithelium like FAE, and where B cells can produce secretory IgA [57]. This further supports the localization of infected tissue in the FAE, where elevated levels of IgA are produced during persistent infection, even though we have also observed strong inhibitory signals, which may be due to the prolonged antigen presence.

#### T-cell response.

The cellular immune response by CD3+ and CD8 + T cells has been suggested to be a driving force for the clearance of persistent FMDV infection in the FAE [58]. The presence of T cells in FMDV-positive samples is indicated by the upregulated T-cell antigen genes *CD4*, *CD83*, *CD84* and *CD86*. From the 20 most upregulated genes of the T-cell activation pathway, 12 genes are associated with negatively regulating T-cell responses (*NLRC3*, *CTLA4*, *CCL19*, *CCR7*, *IKZF3*, *RHOH*, *EGR3*, *LAG3*, *BOLA-DOB*, *TOX*, *BATF*, *SIT1*). The individual functions of several of these genes are listed in **Table 4**, among them *NLRC3*, *IKZF3*, *RIPOR2* and *GRAP*, which were also upregulated on the protein level.

**Table 4:** Genes associated with the suppression of T-cell–mediated immune responses that were significantly upregulated in highly FMDV-positive DSP tissue.

Gene	*p-value*	log2FC	Function	Reference
*NLRC3*	1.58E-03	5.11	Attenuates CD4 + T cell response and autoimmunity	[101]
*TIMD4*	2.47E-04	4.70	Tim-4 + cavity-resident macrophages impair anti-tumor CD8 + T cell immunity	[102]
*CCL19*	4.49E-05	4.33	CCL19/CCR7 drives T_reg_ cell migration	[103]
*CTLA4*	4.66E-02	4.04	Suppresses T cell response	[104]
*CCR7*	2.36E-02	3.71	CCL19/CCR7 drives T_reg_ cell migration	[103]
*RIPOR2* (*FAM65B*)	1.10E-02	3.53	Inhibits chemokine-induced T cell response	[105]
*IKZF3*	2.38E-02	3.43	Deficiency promotes T cell activation	[44]
*RHOH*	4.27E-02	3.30	Impairing T cell chemotaxis	[61]
*EGR3*	8.67E-03	3.29	Suppressive activity to CD4 + T cells and regulates the production of inhibitory cytokines such as IL10 and TGF-β1	[106]
*TOX*	1.35E-02	3.17	Central regulator of T cell exhaustion	[107]
*GRAP*	1.22E-02	3.16	Negatively regulates TCR induced proliferation	[108]
*LAG3*	4.44E-02	3.15	Inhibits the immune microenvironment	[109]
*BATF*	9.63E-03	3.11	Positively regulates Treg cell expression	[110]
*BOLA-DOB*	4.27E-02	3.10	Suppresses antigen loading of MHCII	[76]
*SIT1*	2.20E-02	2.93	Overexpression downmodulates T cell receptor activation	[111]

These genes were identified through differential expression analysis comparing highly FMDV-positive DSP samples with FMDV-negative DSP tissue.

The most upregulated genes that promote T-cell activation, such as *CCL19*, *CCR7* and *ITGAL*, are important for chemotaxis. However, *CCL19* and its specific receptor *CCR7* can play an ambiguous role by mediating the homing of lymphocytes but also by inducing immune tolerance and the recruitment of T_reg_ cells [59], while *ITGAL* facilitates adhesion of T cells to intercellular adhesion molecule-1 and −2 (ICAM1, −2) on endothelia or other T cells [60]. But this effect on chemotaxis and cell adhesion can be antagonized by an upregulation of *RHOH* [61], which was also observed in our samples.

*PD-1*, *CTLA4* and *LAG-3* are important immune checkpoints for regulating T-cell proliferation and their upregulation can occur during chronic viral infections which result in a dysfunctional population of T cells, so-called exhausted T cells. The presence of exhausted T cells in infected tissue is indicated by the upregulation of *CD83* and *CCR7CT* [62]. This T-cell exhaustion allows viruses such as HIV-1 and HCV to persist and is facilitated by inhibitory receptors including PD-1, CTLA4 and LAG-3 [63,64]. The expression of the respective coding genes was significantly positively correlated to FMDV genome load (Fig 4B) and *CTLA4* and *LAG-3* were also significantly differently expressed*.*

IL4I1, which was upregulated on the protein level, can induce the expression of *PD-L1*, a PD-1 ligand [65]. *BATF*, whose expression is directly upregulated by PD-1 and inhibits T-cell proliferation [66], was upregulated as well (see Table 4). T-cell exhaustion is also associated with an overexpression of *MAP4K1* [67] and the immunosuppressive interleukin *IL10*, which were both highly upregulated in FMDV-infected DSP samples. IL10 has been shown to facilitate persistent LCMV infection in rodents [68] and its gene expression was previously observed to be upregulated in FMDV carrier animals [5,11].

Another well-characterized mechanism of inhibiting the T-cell response is the presence of FOXP3 + CD4 + T_reg_ cells. The presence of this special subset of T cells in FMDV-positive DSP samples is indicated by the expression of several marker genes for T_reg_ cells including *FOXP3*, *CTLA4*, *IL2RA* and *TNFRSF18* [69], of which only *CTLA4* was significantly upregulated.

However, gene expression of *FOXP3*, *IL2RA*, and *TNFRSF18* showed a positive correlation to FMDV genome loads (Fig 4C). On the protein level, we detected an upregulation of IL4I1, which inhibits T-cell proliferation and induces T_reg_ cell activation [70]. In accordance with the association of IL10 with T-cell exhaustion described above, T_reg_ cells are a source for this immunosuppressive cytokine [71]. T_reg_ cells prevent lymphocyte migration into affected tissue by the downregulation of neutral sphingomyelinase 3 (*SMPD3*) in endothelial cells [72], which was strongly downregulated in FMDV-positive DSP samples.

In addition to the overexpression of genes that exert a negative influence on the T-cell response in FMDV-positive DSP tissues, some genes positively correlated with T-cell activity were significantly downregulated. Among these, FABP4 was most strongly affected in its gene expression and showed a strong downregulation in its protein expression as well. Together with FABP5, these fatty-acid-binding proteins have been shown to be required for the survival of tissue-resident memory CD8 T cells after viral infection. These cells are located in the epithelial barrier tissue and mediate a first-line response against viral reinfection [73].

For the recognition of pathogens by CD4 T cells, major histocompatibility receptors (MHCII) are a crucial cross-link between antigen-presenting cells (APCs) and CD4 T cells, ultimately leading to the activation of cytotoxic CD8 T cells and the proliferation of T memory cells [74]. Several genes associated with bovine MHCII are significantly upregulated in FMDV-positive DSP samples. Among these the highest upregulation was observed for the *DSB* gene (MHC class II antigen DS beta), an ortholog to *HLA-DRB1* and a structural component of the MHCII receptor. On the protein level, structural components of the MHCII receptor such as BOLA-DRB and SCIMP, which is involved in MHCII signaling [75], were upregulated on gene level as well. The expression of MHCII complexes can be initiated by γ-interferon (IFN-γ) or by the transcription factor CIITA [74], whose coding gene was significantly upregulated. The overexpressed gene *BOLA-DMB*, an ortholog to *HLA-DMB*, is responsible for antigen loading of the MHCII but can be antagonized competitively by *HLA-DO* [76], an ortholog of *BOLA-DO*. Its subunit BOLA-DOB was upregulated to a higher degree than BOLA-DMB on the gene level.

Taken together, this gene expression pattern of major parts of the adaptive immune response shows signs of a prolonged stimulation, which is counteracted by a plethora of differentially expressed genes inhibiting the migration of active cells into the tissue. At same time, an immunologically dysfunctional microenvironment with T_reg_ cells and exhausted T cells is established in the infected FAE.

### Innate Immunity

#### IFN response.

Whether viral infections provoke an antiviral innate immune response depends on the recognition of viral components such as viral RNA or proteins. This leads to an interferon (IFN) response mediated by several signal transduction pathways. FMDV has evolved several inhibitory mechanisms to counteract the IFN response [77]. In this analysis, neither IFN-α nor IFN-β were significantly enriched and only a few genes associated with both pathways were differentially expressed. Only genes responsible for the positive regulation of the IFN-γ pathway were upregulated in FMDV-positive DSP tissue, with overexpression of *LTA*, *TNF*, *SLAMF6*, *SASH3*, *TLR8*, *TLR7*, *IRF8* and *RASGRP1*. On the other hand, the highly overexpressed genes *IL10* and *CCR7* can exert a negative influence on IFN-γ and suppress its expression [78,79]. IFN-γ-induced genes like *STAT1*, *CXCL10*, *CXCL16* and *IFI16* were not significantly upregulated. Only the upregulation of *STAT1* was also observed on the protein level. Even though we and others observed a upregulation of *TNF* [58,80], which could induce an IFN-γ response [81], we did not detect a significant upregulation of genes further downstream in the IFN signaling cascade. This could indicate an inhibition of this pathway, like it was observed by Zhu *et al.* [11], who suggested the activation of the noncanonical NF-κB pathway in carriers.

#### Toll-like receptor (TLR) pathway.

**Fig 5:**
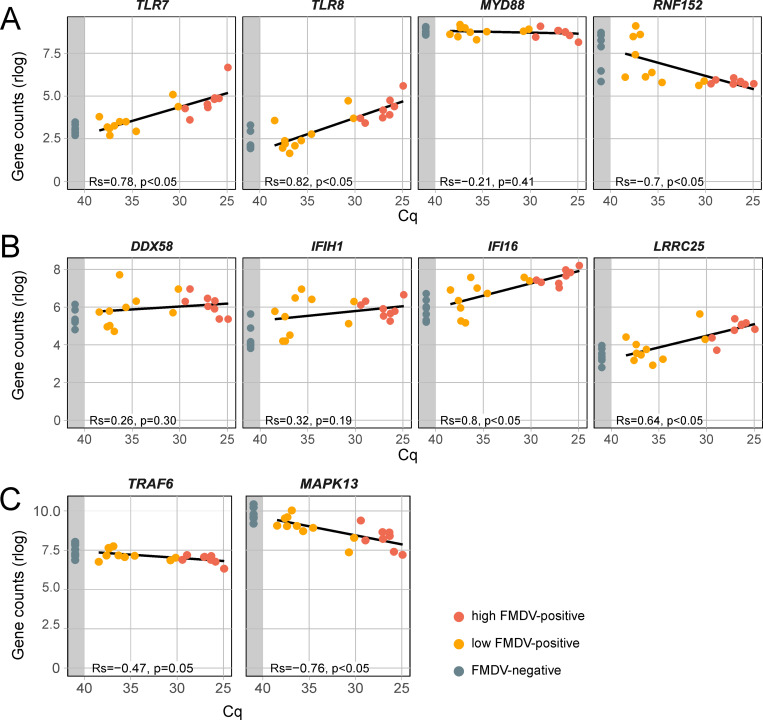
Gene expression of selected genes of the innate immune response signalling cascade in DSP samples. **(A)** Genes of the TLR pathway including *TLR7*, *TLR8*, *MYD88* and *RNF152*. **(B)** Genes involved in the detection of viral RNA: the RNA-sensing receptors *RIG-I* and *MDA5*, *IFI16*, which exerts a positive influence on RIG-I expression, as well as *LRRC25*, possibly upregulated by FMDV 3A and exerting a negative influence on *RIG-I* and *MDA5* expression. **(C)** Downregulated genes downstream the TLR, RLR and NLR pathways including *TRAF6* and *MAPK13* expression. Samples are grouped by their FMDV Cq value into highly positive (Cq ≤ 30, red), weakly positive (Cq > 30, orange) and negative (no Cq, grey area). Gene counts were normalized using a regularized log transformation (rlog) and the result of a linear regression is shown. Correlation between normalized gene counts and FMDV genome load was assed using Spearman’s rank correlation coefficient (Rs). Rs ranges from −1 to 1 and values closer to 1 or −1 indicate a strong positive or negative monotonic relationship, respectively. Rs = 0 indicates no monotonic association. A correlation was considered statistically significant if the *p-value* was less than 0.05.

One important pathway of pathogen recognition is the Toll-like receptor (TLR) pathway. For the detection of endosomal viral ssRNA, especially TLR7 and TLR8 are crucial [82]. *TLR7* as well as *TLR8* were significantly upregulated in FMDV-positive DSP samples and their expression positively correlated to FMDV genome load (**Fig 5**). At the start of the FMDV replication cycle, intact virus particles are present in the endosome, but the viral ssRNA is then ejected from the viral capsid through the endosomal membrane into the cytoplasm [83]. The viral ssRNA might not be easily recognized by TLR7 and −8 at this stage, but during the genome replication, FMDV rearranges host membranes into vesicles for replication, wherein recognition through the TLR could also occur [84]. In contrast to the high expression of TLRs in FMDV-positive samples, *MYD88* was expressed to the same extent as in negative samples regardless of the examined tissue (Fig 5A). MYD88 is a central downstream regulator of the TLR pathway and linked directly to TLR7 and TLR8 [85]. But for further transmission of TLR signaling, MYD88 has to be oligomerized in interaction with RNF152 to allow the recruitment of downstream signaling mediators. An RNF152 deficiency has been shown to exert an negative effect on NF-κB activation, which is downstream of the TLR pathway [86]. A similar effect could occur in FMDV-positive DSP samples, where *RNF152* was significantly downregulated and its expression was significantly negatively correlated to FMDV genome load (Fig 5A). Another interesting significantly upregulated gene in the TLR pathway was *TLR10.* This is the only known TLR to exert an anti-inflammatory influence, and its expression is induced by FOXP3 in T_reg_ cells [87,88].

#### RIG-I-like Receptor (RLR) pathway.

The RIG-I-like Receptor (RLR) pathway is an important component of the innate immune response. There was only a small difference in the expression of the main receptors for viral RNA, *DDX58* (RIG-I) and *IFIH1* (MDA5) between FMDV-positive and negative DSP samples (Fig 5B). The expression of RIG-I, which is responsible for detecting cytoplasmic viral RNA, did not change significantly in samples with high viral genome loads and high *IFI16* expression (Fig 5B). IFI16 has been shown to increase RIG-I expression after detecting viral RNA. [89]. This observation may be explained by the (not significant) upregulation of *LRCC25* (Fig 5B). It has been demonstrated that the FMDV protein 3A upregulates the expression of *LRCC25* and, via inhibition of G3BP1, downregulates the expression of RIG-I and MDA5 [90]. This recently discovered mechanism may have an important influence during the persistent infection to allow the continued presence of viral RNA in the cytoplasm in a balance between RIG-I upregulation by IFI16 and downregulation by LRRC25. In a comparative transcriptome study of native Indian and European cattle breeds, increased RIG-I/MDA5-mediated IFN signaling was indeed associated with milder clinical signs during the acute phase of FMD, but persistent infection was not investigated [91].

#### NOD-like receptor (NLR) pathway.

TRAF6 is a central signaling component downstream of the TLR, RLR and NOD-like receptor (NLR) pathways leading to NF-κB activation amongst others [77]. In DSP, *TRAF6* expression is negatively correlated to FMDV genome load (Fig 5C). It is targeted by the FMDV leader protease L^pro^, which deubiquitinates and thereby inactivates TRAF6 [92]. A direct regulation of its expression has not been demonstrated, but deubiquitinating TRAF6 could cause a negative feedback loop [93]. The downregulation of *TRAF6* has previously been observed in a microarray study with tissue samples of persistently FMDV-infected animals [10]. MAPK13 is a not well characterized p38 isoforms, which is activated by TRAF6 [94] and could contribute to the inhibition of apoptosis. In the DSP, the *MAPK13* gene expression is significantly negatively correlated to FMDV genome load (Fig 5C).

### Lysozyme

The important antimicrobial protein lysozyme is usually found in lymphoid tissue and lymphoid epithelia as an effective agent against invading microbes [95]. Two bovine genes associated with lysozyme were significantly downregulated in FMDV-positive samples: *LYZ1* and LOC112446693 coding for a tracheal isozyme-like lysozyme C. This is of particular interest since lysozyme is usually secreted in epithelia for protection and can exert limited antiviral activity [96].

### Phospholipases

Phospholipases are a part of the cellular lipid metabolism but exert important immunological functions beyond that. Several genes coding for phospholipases were differentially expressed in FMDV-positive DSP samples. Most of the differentially expressed genes belong to the phospholipase A2 (PLA2) family and can be categorized into specific subfamilies [97], particularly cytosolic PLA2 (cPLA2) and secreted PLA2 (sPLA2). The sPLA2s act extracellularly to generate lipid mediators involved in inflammation and host defense. They can be pro- or anti-inflammatory depending on the context. The cPLA2s, on the other hand, work intracellularly by releasing arachidonic acid and thus promoting inflammation.

Genes of the cPLA2 subfamily (*PLA2G4B* and *PLA2G4F*) were significantly downregulated. These are associated with inflammatory skin diseases and, for PLA2G4B in particular, it has been shown that it promotes the expression of other inflammatory genes such as *IL36A* and *IL36G* [98]. Fittingly, in our FMDV-positive samples, *IL36A* and *IL36G* were significantly downregulated as well. In contrast, a phospholipase of the sPLA2 subfamily (*PLA2G2D4*) was significantly upregulated on the gene level, and PLAG2a of the same subfamily was also upregulated on the protein level. Among the sPLA2 subfamily, the *PLA2G2D4* gene is expressed by dendritic cells and macrophages and supports the clearance of inflammation [99].

Another phospholipase relevant to this study is phospholipase C, whose encoding gene *PLCG2* was also significantly upregulated. Phospholipase C has diverse functions: On the one hand, it facilitates the survival of mature B cells and antibody production in the adaptive immune system. On the other hand, it acts as a downstream signalling molecule after Fc receptor (FcR) activation, provoking an antibacterial response in the innate immune system [100].

The phospholipases are a diverse group and their functions are similarly diverse. The expression levels of the genes mentioned in this paragraph can be found in S3 Table. Particularly the distribution of PLA2 describes an environment in which inflammation is resolved by the upregulation of anti-inflammatory sPLA2s in the extracellular compartment and by the downregulation of intracellular proinflammatory cPLA2s.

## Conclusions

The animal experiment that this study is based on resulted in a large number of carrier animals with a high incidence of persistent infection. This allowed for the first time ever a simultaneous analysis of the transcriptome and proteome of *in vivo* tissue samples from persistently FMDV-infected animals. Furthermore, the use of the same samples for transcriptomic and proteomic analysis allowed us to corroborate our findings within our own study.

In our cohort of 18 animals, regardless of their vaccination status, the DSP tissue contained a higher proportion of FMDV-positive samples and higher viral genome loads than the DNP tissue, which has often been described as the preferential site of persistent FMDV infection [4]. However, we did confirm that persistent FMDV is likely found in the FAE, as previously demonstrated with other methods, such as LCM or immunofluorescence [5]. The observed differences between FAE and adjacent epithelial, uninfected cells in the expression of genes associated with the extracellular matrix, cell-to-cell connections and desquamation, may explain the enhanced susceptibility of the FAE for persistent infection.

Between survival and death, the fate of cells is decided by the balance of differentially expressed genes with pro- and anti-apoptotic functions. An enhanced resistance to apoptosis in tissues susceptible to persistent FMDV infection has already been suggested [33]. Based on our results, the strong expression of *BCLA1* is likely to play a decisive role in regulating apoptosis. This upregulation of *BCLA1* has previously been described in FMDV carriers compared to non-carriers [9]. Additionally, the anti-apoptotic function of SPIB which is expressed in M-cells of the FAE [29] hints at a tissue-specific advantage of the FAE that prevents the demise of its epithelial cells.

It is well known that there is a strong FMDV-specific IgA response in secretions of carrier animals and that this can even be exploited for the diagnosis of persistent infection [52]. In our set of samples, we observed a gene expression pattern that suggests the presence of IgA-producing B cells in lymph follicles underlying the FAE, but inhibitory signals were present as well. More revealing of the virus-host relationship is the gene expression pattern influencing the T-cell response. We observed both T-cell activation and repression at the same time. However, genes and proteins exerting a negative influence on T cells seem to be dominating (see **Table 4).** This transcriptomic and proteomic landscape paints a picture of a microenvironment in the FAE and the underlying lymph follicles favoring T-cell exhaustion and the presence of T_reg_ cells, while inhibiting efficient clearance of virus by cytotoxic T cells. This cellular immune response has previously been hypothesized to be responsible for the inability of carrier animals to clear FMDV from the nasopharynx [9,58].

Concerning the innate immune response, we noted an absence of differentially expressed genes associated with an IFN type I response. The IFN-γ pathway was found to be activated, but ISGs were not significantly overexpressed. Several genes involved in TLR and RLR pathways were downregulated in FMDV-positive samples, which could be caused by the IFN-inhibitory functions of FMDV. Of particular interest is the upregulation of *LRRC25*, indicating an inhibition of RIG-I and MAPK5 which can be caused by the viral FMDV protein 3A [90], as well as the downregulation of *TRAF6*, which is targeted by FMDV L^pro^ [92]. The inhibition of innate immune responses following detection of viral RNA via the TLR, RLR and NLR pathways may be actively facilitated by FMDV viral proteins targeting central signalling components such as RIG-I, MDA5 and TRAF6. Major pressure on the type I IFN response is exerted by the FMDV leader protease L^pro^ and this might be a critical factor in the establishment of persistent infection. Leaderless FMDV are strongly attenuated and unable to persist in cattle [24].

Ultimately, the reason for the preferential localisation of persistent FMDV infection in the FAE remains elusive. This microanatomic compartment, which is also the location of primary replication of FMDV in cattle, serves as a reservoir of FMDV in an otherwise immune host. We suspect that the persistent infection of FMDV in the epithelia of the nasopharynx is enabled by an interplay between virally induced inhibition of the innate immune response, suppression of apoptosis and a permissive microenvironment of T_reg_ cells and exhausted T cells in a highly susceptible epithelium.

## Supporting information

S1 Fig Top 10 canonical pathways sorted by *p-value.* A right-tailed Fisher’s Exact Test was used to calculate a *p-value* determining the probability that the association between the genes in the dataset and the canonical pathway is explained by chance alone. Bars represent –log10(*p-value*), with the horizontal threshold line indicating the significance cutoff. The color of each bar reflects the predicted activation state derived from the pathway Z-score: orange bars indicate predicted activation (positive Z-score), blue indicates predicted inhibition (negative Z-score), white indicates pathways with no predicted activation state (Z-score = 0) and grey indicates insufficient data to compute an activity pattern.
(TIF)

S2 Fig Integrated pathway analysis of the apoptosis canonical pathway. Solid lines are direct relationships and dashed lines are indirect. Purple outline = measured as differentially expressed – intensity of colored infill indicating the level of up (red) or down (green) regulation, blue color = predicted inhibition, orange = predicted activation (Molecular Activity Predictor function), Yellow lines = inconsistency with the state of the downstream molecule. Functional classes: nested circle/square = group/complex, horizontal ellipse = transcriptional regulator, vertical ellipse = transmembrane receptor, vertical rhombus = enzyme, square = cytokine/growth factor, triangle = kinase, vertical ellipse = transmembrane receptor, circle = other.
(TIF)

S1 Table Tissue samples collected during necropsy submitted for RNAseq transcriptomic analysis. (XLSX)

S2 Table Differentially expressed genes. (A) Comparing all DSP to all DNP samples. (B) Comparing FMDV-negative (no Cq) DSP to DNP samples. (C) Differentially expressed genes when comparing highly positive FMDV (Cq ≤ 30) to FMDV-negative (no Cq) DSP samples.
(XLSX)

S3 Table The differential expression of genes associated with desquamation of superficial cells was investigated. Highly FMDV-positive (Cq ≤ 30) DSP samples were compared with FMDV-negative samples (no Cq).
(XLSX)
