# Supplementary figures and images for: Persistent foot-and-mouth disease virus infection of the bovine nasopharynx is associated with suppression of innate and cellular immunity
Source: PLoS One. 2026 Jan 23;21(1):e0340425. doi: 10.1371/journal.pone.0340425 (PMC12829788; doi:10.1371/journal.pone.0340425)

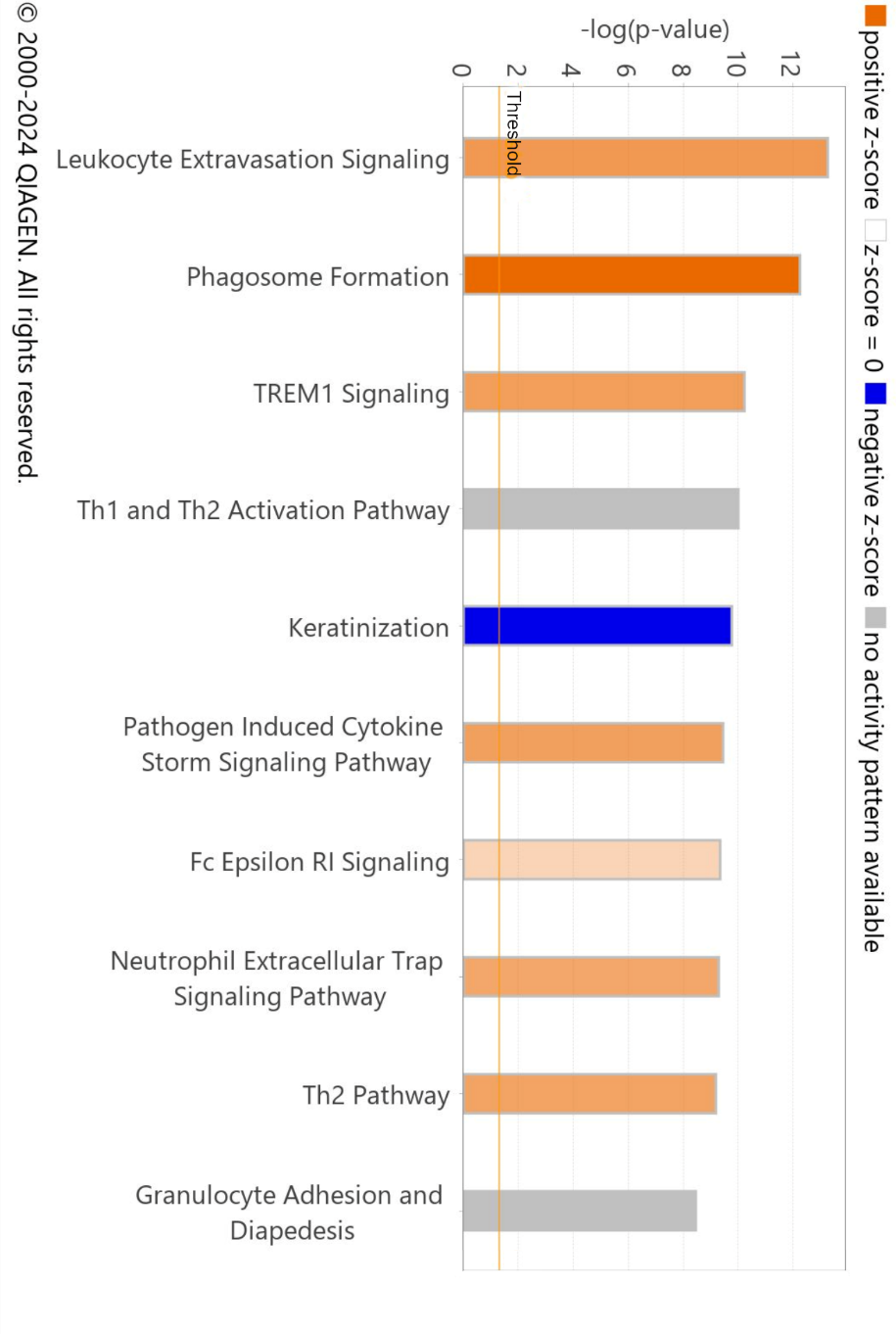

Supplement: S1 Fig — A right-tailed Fisher’s Exact Test was used to calculate a p-value determining the probability that the association between the genes in the dataset and the canonical pathway is explained by chance alone. Bars represent –log10(p-value), with the horizontal threshold line indicating the significance cutoff. The color of each bar reflects the predicted activation state derived from the pathway Z-score: orange bars indicate predicted activation (positive Z-score), blue indicates predicted inhibition (negative Z-score), white indicates pathways with no predicted activation state (Z-score = 0) and grey indicates insufficient data to compute an activity pattern. (TIF) [file pone.0340425.s001.tif]

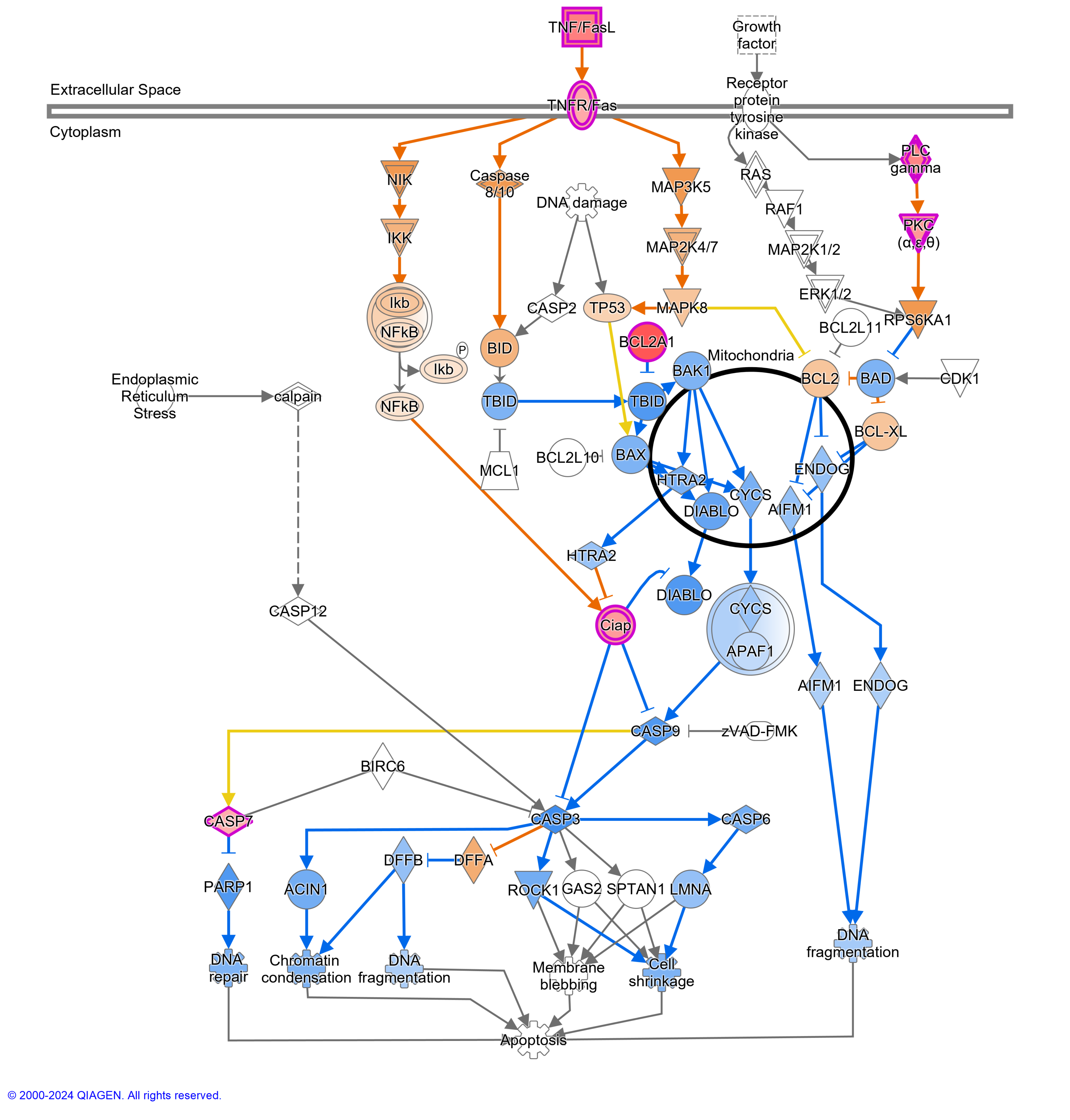

Supplement: S2 Fig — Solid lines are direct relationships and dashed lines are indirect. Purple outline = measured as differentially expressed – intensity of colored infill indicating the level of up (red) or down (green) regulation, blue color = predicted inhibition, orange = predicted activation (Molecular Activity Predictor function), Yellow lines = inconsistency with the state of the downstream molecule. Functional classes: nested circle/square = group/complex, horizontal ellipse = transcriptional regulator, vertical ellipse = transmembrane receptor, vertical rhombus = enzyme, square = cytokine/growth factor, triangle = kinase, vertical ellipse = transmembrane receptor, circle = other. (TIF) [file pone.0340425.s002.tif]
